# Human islet isolation optimization: Insights from donor and isolation procedural factors

**DOI:** 10.1177/09636897261433325

**Published:** 2026-03-24

**Authors:** Qin Yang, Yinsheng Xi, Zhihong Yang, Hongping Deng, Zhenjuan Wang, Guoping Li, Kerry Augusta, Shimul Shah, James F. Markmann, Ji Lei

**Affiliations:** 1 Penn Transplant Institute, Department of Surgery, Hospital of the University of Pennsylvania, Philadelphia, PA, USA; 2 Transplant Division, Center for Transplantation Sciences, Massachusetts General Hospital, Harvard Medical School, Boston, MA, USA

**Keywords:** islets transplantation, diabetes mellitus, type 1, digestion, perfusion, donor selection

## Abstract

This study investigated donor and islet isolation procedural factors influencing the islet yield during human islet isolation for transplantation. We retrospectively analyzed 133 islet isolations from deceased donors performed over 15 years at a single center. Isolations were stratified by post-purification islet yield (≥400,000 islet equivalents [IEQ], successful; <400,000 IEQ, unsuccessful) and by intent (clinical vs research). Higher donor body mass index and height over 170 cm were independently associated with successful isolation, whereas an enzyme perfusion temperature exceeding 14°C for ≥50% of the perfusion duration emerged as an independent risk factor for isolation failure. Clinically intended isolations exhibited tighter thermal control during Phase 1 digestion, greater digestion efficiency, and lower undigested tissue weight. A logistic regression model incorporating body surface area, packed tissue volume, and phase 1 digestion time (the interval from the start of warm recirculation to the collection phase) showed a moderate predictive value for isolation success (area under the curve = 0.755). Subgroup analysis revealed that longer relative phase 2 digestion time (the interval from the start of collection to its end) and higher North American Islet Donor Scores were associated with higher islet yield. These findings highlight the importance of donor anthropometrics, procedural consistency, and thermal regulation during islet isolation. Optimizing donor selection and controlling intra-procedural variables can improve islet yield and increase the likelihood of achieving the required clinical transplantation islet dose.

## Introduction

Human islet transplantation is a critical therapeutic option for individuals with type 1 diabetes (T1D), offering improved glycemic control and, in some cases, independence from exogenous insulin^1–4^. However, the success of this procedure depends heavily on the quality and quantity of isolated islets, which in turn is influenced by multiple donor- and procedure-related variables. As clinical islet transplantation typically requires >5000 IEQ/kg of recipient body weight, we defined successful isolation as a post-purification yield ≥400,000 IEQ, which corresponds to the highest threshold reported in prior studies^5,6^. Under this stringent definition, a single isolation would provide an adequate dose for most recipients weighing <80 kg.

The islet isolation process is technically demanding, requiring sophisticated techniques to optimize conditions for obtaining sufficient viable islets. Donor-specific factors such as body mass index (BMI), donor height, and medical history, along with procedural variables including perfusion temperature and digestion time, have been shown to significantly impact isolation outcomes^7,8^. Organ preservation variables, such as cold ischemia time (CIT), also contribute to variability in islet recovery, though the optimal thresholds remain debated^9–16^.

Despite adherence to standardized protocols, variability in isolation outcomes persists, likely due to complex interactions among donor characteristics, organ quality, and operator-dependent procedural nuances^5,17–19^. A clearer understanding of how these variables influence yield is essential for improving isolation efficiency and ensuring an islet mass suitable for clinical transplantation^20–24^. In this study, we retrospectively analyzed 133 islet isolations performed over a 15-year period at a single center. Our aim was to identify donor and procedural factors associated with successful islet isolation and to define procedural benchmarks that can enhance consistency and maximize yield for clinical application.

## Materials and methods

### Study cohort

We conducted a retrospective analysis of 133 human islet isolation procedures performed at Massachusetts General Hospital between 2007 and 2021. All pancreata were obtained from deceased donors through certified Organ Procurement Organizations (OPOs) under appropriate research consent. No living human subjects were involved. This study involved human islet isolations from deceased donors obtained through certified OPOs. According to institutional policy, research involving deceased donor tissue does not constitute human subjects research and was determined to be exempt from review by the Massachusetts General Hospital Institutional Review Board. All procedures complied with applicable guidelines and regulations governing research using deceased donor tissue.

### Islet isolation protocol

Islet isolation was performed according to the Clinical Islet Transplantation Consortium (CITC) protocol with center-specific modifications^6,25^. Cadaveric pancreata were procured using standard surgical techniques and preserved in cold storage solution immediately after harvest.

After trimming, weighing, and assessing the organs for morphology (fat infiltration, edema, texture, vascular flush, and capsular integrity), the main pancreatic duct was cannulated using a 16- to 18-gauge catheter. Enzyme perfusion was performed for approximately 10 min (≤12 min), at 4–14°C, using volume-adjusted CITC enzyme solution, as specified in the CITC protocol. All isolations were performed using Good Manufacturing Practice (GMP) grade enzyme preparations, including Collagenase NB 1/Neutral Protease NB (SERVA/Nordmark), CIzyme Collagenase HA/Thermolysin (VitaCyte), and Liberase MTF C/T (Roche).

The pancreas was then sectioned into 5–15 similar-sized pieces of 1–2.5 inches in length and placed into a Ricordi digestion chamber. Digestion occurred under enzyme recirculation at 37°C (range 32–38°C), with manual or mechanical agitation (phase 1 digestion). Small tissue samples were assessed microscopically every 2–5 min for islet release, fragmentation (islets with a ragged border due to enzyme overexposure), and digestion status^26^. Phase 1 digestion time was defined as the interval from the start of warm recirculation to the decision point to switch to the collection phase.

Phase 2 digestion was initiated once predefined criteria were met (>45 free islets, >50% free islet fraction, and <10% fragmentation by CITC protocol)^6^. Enzyme activity was then terminated by cooling and dilution, and the digestate was washed. Phase 2 digestion time was defined as the interval from the start of collection to its termination. Packed tissue volume (PTV) was recorded post-centrifugation.

Islet purification was performed using continuous density gradient centrifugation with a COBE 2991 cell processor^27^. Islet yield was expressed in IEQ, standardized to a 150-μm diameter, and viability was assessed by fluorescein diacetate/propidium iodide staining.

Endotoxin levels, Gram staining, and bacterial cultures were obtained per standard protocols.

### Donor and organ variables

Donor variables included age, sex, race, height, weight, BMI, body surface area (BSA; Du Bois formula: BSA = 0.007184 × height^0.725^ × weight^0.425^), cause of death, vasopressor use, medical history, duration of acute illness and brain death, and peak blood glucose, amylase, and lipase levels. Height was analyzed both as a continuous variable and, in a post hoc exploratory analysis, as a categorical variable using a 170-cm cutoff. Organ-related variables included OPO origin (local vs distant), CIT, and the North American Islet Donor Score (NAIDS)^5^. Pancreatectomy time (lukewarm ischemia time) was defined as the interval from aortic cross-clamping with the initiation of cold preservation solution to completion of pancreatectomy. CIT was defined as the interval from aortic cross-clamp time to initiation of enzyme perfusion for islet isolation. Pancreas morphology was evaluated using a composite score (0–12), which was based on fat infiltration, edema, vascular flush, tissue texture, and capsular integrity (see scoring matrix below).

**Table:** Pancreatic morphological scoring matrix.

Parameter	Score		
	0	1	2
Fat infiltration	heavily	patchy	clean or average
Flush	poorly	well	excellent
Blood	capillaries	intra-parenchymal	none
Edema	overly distended	slightly overall swelling	interstitial or none
Texture	rigid throughout or very soft	many firm areas or soft	normal
Overall condition	parenchymal damage	capsular damage	intact

### Procedural variables

Procedural variables included trimmed pancreas weight, digestion time (phase 1 and phase 2), enzyme perfusion temperature, and digestion temperature. The target perfusion temperature was 4–14°C for approximately 10 min, as specified by the CITC protocol. Cases in which the temperature exceeded 14°C for ≥50% of the perfusion duration were classified as the >14°C group. Perfusion temperature was therefore analyzed as >14°C versus ≤14°C to assess the impact of these elevations on islet isolation outcomes. Digestion temperature was categorized as tightly controlled when 37–38°C was maintained for ≥50% of the phase 1 digestion duration.

### Outcome parameters

Primary outcomes included post-purification IEQ, islet particle number (IPN), viability, purity, percentage of free islets, and packed cell volume (PCV). Purity was calculated as a weighted average across high (≥70%), middle (40–69%), and low (30–39%) fractions. As clinical islet transplantation typically requires a dose exceeding 5000 IEQ per kilogram of recipient body weight, we defined successful isolation as a post-purification yield ≥400,000 IEQ, which corresponds to a commonly used threshold in previous studies^5,6^. Under this stringent definition, a single isolation would provide an adequate dose for most recipients weighing <80 kg.

### Statistical analysis

Continuous variables were first tested for normality using the Kolmogorov–Smirnov test. Variables with normal distribution are presented as mean ± SD and compared using the independent samples *t* test, whereas non-normally distributed variables are presented as median (interquartile range [IQR]) and compared using the Mann–Whitney U test. Categorical variables are presented as percentages and analyzed using Fisher’s exact test or chi-square test. Correlation between NAIDS and post-purified islet yield was assessed using both Spearman’s rank correlation and Pearson’s correlation. As NAIDS and IEQ were not normally distributed, Spearman’s rho was considered the primary analysis, while Pearson’s correlation was reported for comparison, given the presumed linear relationship between the variables. Univariate and multivariate logistic regression identified the predictors of islet yield ≥400,000 IEQ and intent-to-treat clinical transplantation. A multivariable logistic regression model was constructed to identify predictors of successful islet isolation. Candidate variables included BSA, PTV, and phase 1 digestion time, which were selected based on biological plausibility and univariable screening. Model performance was assessed using receiver operating characteristic (ROC) analysis, with discrimination quantified by the area under the curve (AUC) and 95% confidence interval. A two-sided *P* value <0.05 was considered statistically significant. All analyses were conducted using SPSS Statistics version 31 (IBM, Armonk, NY, USA).

## Results

### Donor and organ characteristics

A total of 133 deceased donor islet isolations were included in this analysis. The demographics of the donors are shown in Table 1. The mean donor age was 46.3 ± 12.5 years, and 60.8% were male. Most donors were white (82.1%), and cerebrovascular accidents account for 48.5% of deaths. Most organs (92.5%) were recovered by distant OPOs. The most common blood type was O (45 %). The median CIT was 8.1 h (IQR, 6.7–9.7). Median peak amylase and lipase levels were 86 U/L and 28 U/L, respectively. Median maximum and minimum blood glucose levels were 225 mg/dl and 103 mg/dl, respectively. Mean BMI was 31.4 ± 7.1 kg/m^2^, and the mean BSA was 2.1±0.3 m^2^. BSA showed only a modest correlation with trimmed pancreas weight (Spearman rho = 0.322, *P* < 0.001).

**Table 1.** Demographics of pancreas donors.

	Total (n = 133)	≥400k IEQ (n = 58)	<400k IEQ (n = 75)	*P*	Size effect
Age (years)	46.3 ± 12.5	46.7 ± 11.4	46.1 ± 13.3	0.77	
Sex, male, n (%)	79 (60.8)	37 (66.1)	42 (56.8)	0.28	
Race				0.49	
*White/Caucasian, n (%)*	101 (82.1)	45 (83.3)	56 (81.2)		
*Black/AA, n (%)*	13 (10.6)	4 (7.4)	9 (13)		
*Other, n (%)*	9 (7.3)	5 (9.3)	4 (5.8)		
Height (cm)	173.4 ± 9.5	175.2 ± 9.7	172.0 ± 9.2	0.05	
≥170 cm	85 (63.9)	45 (77.6)	40 (53.3)	0.004	
Weight (kg)	93.9 ± 24.0	100.9 ± 23.0	88.5 ± 23.5	0.003	0.6
BMI (kg/m^2^)	31.4 ± 7.1	33.2 ± 7.6	30.0 ± 6.5	0.008	0.5
BSA (m^2^)	2.1 ± 0.3	2.1 ± 0.2	2.0 ± 0.3	<0.001	0.7
OPT, distant, n (%)	123 (92.5)	55 (94.8)	68 (90.7)	0.37	
Cause of death				0.96	
*Anoxia, n (%)*	27 (20.5)	13 (22.4)	14 (18.9)		
*Cerebrovascular, n (%)*	65 (48.5)	27 (46.6)	37 (50)		
*Trauma & others, n (%)*	41 (31.0)	18 (31.0)	23 (31.1)		
Types of vasopressors				0.79	
*None, n (%)*	29 (27.1)	12 (26.1)	17 (27.9)		
*Single, n (%)*	64 (59.8)	29 (63)	35 (57.4)		
*Two or more, n (%)*	14 (13.1)	5 (10.9)	9 (14.8)		
Medical history					
*Insulin therapy, n (%)*	63 (51.2)	31 (59.6)	32 (46.4)	0.15	
*Alcohol intake, n (%)*	89 (70.6)	40 (74.1)	49 (68.1)	0.46	
*Smoking, n (%)*	64 (50.8)	22 (40.7)	42 (58.3)	0.05	
*Hypertension, n (%)*	44 (34.9)	22 (40.7)	22 (30.6)	0.24	
*Cardiac arrest, n (%)*	28 (23.7)	12 (23.1)	16 (24.2)	0.88	
*Respiratory arrest, n (%)*	16 (14.2)	5 (10.2)	11 (17.2)	0.29	
*Hypotension, n (%)*	28 (24.3)	10 (19.2)	18 (28.6)	0.25	
*Cytomegalovirus, n (%)*	61 (48)	27 (48.2)	34 (47.9)	0.97	
Blood type				0.53	
*A*	50 (38.8)	25 (44.6)	25 (34.2)		
*B*	14 (10.9)	4 (7.1)	10 (13.7)		
*O*	58 (45)	24 (42.9)	34 (46.6)		
*AB*	7 (5.2)	3 (5.4)	4 (5.5)		
Minimum BGL (mg/dL)	103 (90–119)	110 (94–124)	104 (91–127)	0.52	
Maximum BGL (mg/dL)	225 (187–278)	202 (175–262)	227 (194–285)	0.12	
Maximum lipase (U/L)	28 (20–86)	35 (23–115)	23 (17–60)	0.04	0.8
Maximum amylase (U/L)	86 (50–197)	72 (47–268)	107 (61–197)	0.95	
Acute illness time (day)	1 (1–4.5)	1 (1–1.5)	1 (1–4)	0.54	
Brain death time (h)	25 (16.1–36)	17 (14–32)	14 (10–27)	0.18	
Cold ischemia time (h)	8.1 (6.7–9.7)	8.6 (7.2–10.5)	8.8 (6.6–10.6)	0.66	
NAIDS	69 (57.3–82)	75 (64–86)	62 (55–74)	<0.001	0.6
Morphology score	8 (8–11)	8 (7–11)	8 (6.5–9.5)	0.93	
Trimmed pancreas (g)	96 (83–110)	98 (86–110)	91 (82–101)	0.097	

Categorical variables were analyzed by Fisher exact test. Continuous variables were first tested for normality using the Kolmogorov–Smirnov test. Variables with normal distribution are presented as mean ± SD and compared using the independent samples *t* test, whereas non-normally distributed variables are presented as median (IQR) and compared using the Mann–Whitney U test. IQR, interquartile range; AA, Africa-American; OPT, organ procurement team; BMI, body mass index; BSA, body surface area; BGL, blood glucose level; NAIDS, North American Islet Donor Score; IEQ, islet equivalent number; 400K, 400,000; ns, nonsignificant. A value of *P* < 0.05 was considered significant.

When stratified by post-purification islet yield (≥400,000 IEQ [successful] vs <400,000 IEQ [unsuccessful]), no significant differences were observed in age, sex, race, OPO origin, cause of death, vasopressors use, medical history, blood type, glucose levels, peak amylase levels, duration of illness or brain death, CIT, or morphology score (all *P* > 0.05). However, the successful group exhibited significantly higher body weight (mean 100.9 kg vs 88.5 kg, *P* = 0.003), BMI (mean 33.2 kg/m^2^ vs 30.0 kg/m^2^, *P* = 0.008), BSA (mean 2.1 m^2^ vs 2.0 m^2^, *P* < 0.001), peak lipase (median 35 U/L vs 23 U/L, *P* = 0.04), and NAIDS (median 75 vs 62, *P* < 0.001). Height did not differ significantly between groups when analyzed as continuous (mean 175.2 cm *vs.* 172.0 *cm*, *P* = 0.05). In a post hoc exploratory analysis, a greater proportion of the successful group were taller than 170 cm compared with the unsuccessful group (77.6% vs 53.3%, *P* = 0.004).

### Procedure parameters

Variables related to the islet isolation procedure are displayed in Table 2. Comparing to the unsuccessful group, successful isolations had significantly greater use of University of Wisconsin (UW) solution (100% vs 77%, *P* < 0.001) and shorter phase 1 digestion time (median 16 min vs 17 min, *P* = 0.002). Compared with the unsuccessful group, the successful group had a significantly larger proportion of cases with enzyme perfusion temperatures maintained within 4–14°C (91.1% vs 68.1%, *P* = 0.002) and with tightly controlled digestion temperatures (37–38°C for ≥50% of phase 1 digestion duration; 81% vs 60.8%, *P* = 0.01). Cases in which the perfusion temperature exceeded 14°C for 50% of the perfusion duration were analyzed as a distinct >14°C group to assess the potential effect of these elevations on isolation outcomes. Other procedural variables, including enzyme type and amount and phase 2 digestion time, did not differ significantly between groups.

**Table 2.** Comparison of islet isolation procedure variables between groups based on post-purified yield (yield ≥400,000 IEQ vs <400,000 IEQ).

	Total (n = 133)	≥400k IEQ (n = 58)	<400k IEQ (n = 75)	*P* value	Size effect
NP, SERVA, n (%)	67 (52.3)	29 (51.8)	38 (52.8)	0.91	
Col, SERVA, n (%)	65 (50.8)	27 (48.2)	38 (52.8)	0.61	
Preserve solution, UW, n (%)	97 (87.4)	50 (100)	47 (77)	<0.001	0.3
Neutral protease (U/V) ×10^3^	0.3 (0.2–166)	0.3 (0.2–170)	0.3 (0.2–158)	0.07	
Collagenase (U/V) ×10^3^	2.7 (2.1–3.1)	2.6 (2.3–2.8)	2.5 (1.7–3.1)	0.32	
Concentration of NP (units/ml)	0.8 (0.6–474)	0.8 (0.7–486)	0.8 (0.6–453)	0.14	
Concentration of Col (units/ml)	7.6 (5.3–8.8)	7.3 (6.3–7.9)	7.3 (4.6–8.8)	0.26	
NP/trimmed (units/g) ×10^3^	1.6 (2.4–2143)	3.1 (2.4–1981)	3.2 (2.4–1827)	0.41	
Col/trimmed tissue (units/g)	28.1 (20.7–32.3)	25.7 (22.4–30.0)	28 (19.4–34.0)	0.56	
Digestion time (phase 1, min)	16 (13–19)	16 (12.5–17.5)	17 (14.5–19.0)	0.002	0.5
Digestion time (phase 2, min)	21 (14–26)	21 (15–27)	23 (16.5–26.5)	0.47	
Perfusion temperature, n (%)				0.002	0.3
*4–14°C*	100 (78.1)	51 (91.1%)	49 (68.1%)		
*>14°C*	28 (21.9)	5 (8.9%)	23 (31.9%)		
Digestion temperature, n (%)				0.01	0.2
*37–38°C*	92 (69.7)	47 (81)	45 (60.8)		

Categorical variables were analyzed by Fisher exact test. Continuous variables were first tested for normality using the Kolmogorov–Smirnov test. Variables with normal distribution are presented as mean ± SD and compared using the independent samples *t* test, whereas non-normally distributed variables are presented as median (IQR) and compared using the Mann–Whitney U test. IQR, interquartile range; NP, neutral protease; COL, collagenase; IPN, islet particle number; IEQ, islet equivalent number; UW solution, University of Wisconsin solution; HTK solution, Histidine-Tryptophan-Ketoglutarate solution; NAIDS, North American Islet Donor Score; PTV, packed tissue volume; PCV, packed cell volume; 400K, 400,000; ns, nonsignificant. A value of *P* < 0.05 was considered significant.

### Isolation outcomes

Key outcome metrics are summarized in Table 3. The successful group demonstrated significantly higher PTV, higher purified IEQ, total IPNs, and percent islet recovery. Viability, purity, and percentage of free islets were comparable between groups.

**Table 3.** Outcomes of islet isolation between isolations with versus without post-purification yield ≥400,000 IEQ.

	Total (n = 133)	≥400k IEQ (n = 58)	<400k IEQ (n = 75)	*P* value
Digested tissue (g)	18.2 (12–33)	15 (11.5–21.5)	20 (13–34)	0.22
PTV (ml)	40 (30–45)	45 (40–50)	38 (24–48)	0.001
Pre-purified IPNs×10^3^	570 (371–1097)	820 (719–1156)	375 (296–575)	<0.001
Pre-purified IEQ×10^3^	612 (327–790)	724 (646–861)	317 (241–442)	<0.001
Free islet percentage (%)	70 (60–80)	60 (55–75)	70 (50–90)	0.06
Pre-purified islet/trimmed (IEQ×10^3^/g)	5.7 (3.3–8.7)	7.9 (5.7–9.4)	3.9 (2.7–4.3)	<0.001
Pre-purified islet/digested (IEQ×10^3^/g)	7.8 (5.2–11.4)	8.9 (7.5–11.3)	5.0 (3.5–7.2)	<0.001
Purified IPN×10^3^	370 (242–704)	724 (550–895)	260 (145–358)	<0.001
Purified IEQ×10^3^	409 (187–676)	646 (476–770)	214 (116–272)	<0.001
High fraction IEQ (%)	72 (47–86)	74 (60–86)	77 (39–100)	0.56
High fraction IEQ ×10^3^	230 (114–504)	486 (309–604)	142 (28–223)	<0.001
High fraction IPN×10^3^	289 (117–513)	518 (423–707)	222 (64–304)	<0.001
Purified islet/trimmed tissue				
*×10*^3^*IEQ/g*	3.3 (1.9–7.0)	7.0 (5.2–8)	2.2 (1.1–3)	<0.001
*×10*^3^*IPN/g*	4.1 (2.3–7.7)	7.9 (6.0–9.1)	2.6 (1.6–4.6)	<0.001
Purified islet digested tissue				
*×10*^3^*IEQ/g*	5.2 (2.7–8.5)	8.3 (6.6–9.4)	2.9 (1.7–3.8)	<0.001
*×10*^3^*IPN/g*	5.6 (3.0–9.1)	9.0 (7.2–10.6)	3.1 (2.1–5.6)	<0.001
Islet recovery (%)	71.6 (41.6–92.6)	96 (83–100)	45 (37–876)	<0.001
High fraction viability	95 (95–99)	95 (95–99)	95 (95–95)	0.001
Average viability	95 (95–99)	95 (52–99)	95 (93–98)	0.51
Average purity	68.7 (56.9–79.7)	68 (58.5–78)	63 (48–79)	0.75
PCV (ml)	2.1 (1.3–3.6)	3.4 (2.2–3.8)	1.3 (0.8–2.1)	<0.001
High fraction PCV (%)	48.6 (33–65)	47 (38–60)	55 (33–100)	<0.001

Categorical variables were analyzed by Fisher exact test. Continuous variables were first tested for normality using the Kolmogorov–Smirnov test. Variables with normal distribution are presented as mean ± SD and compared using the independent samples *t* test, whereas non-normally distributed variables are presented as median (IQR) and compared using the Mann–Whitney U test. IQR, interquartile range; IPN, islet particle number; IEQ, islet equivalent number; PCV, packed cell volume; 400K, 400,000; ns, nonsignificant. A value of *P* < 0.05 was considered significant.

### Correlation and regression analyses

**Figure 1.**
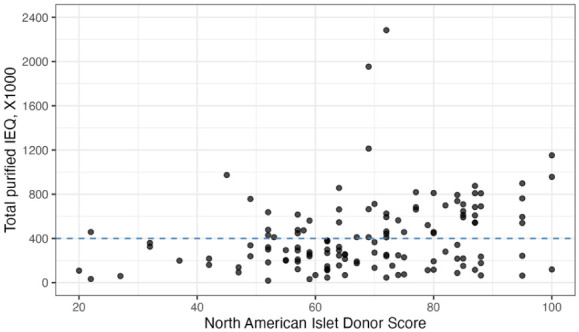
Correlation between NAIDS and post-purification islet yield. Scatterplot showing the relationship between NAIDS and post-purification IEQ. NAIDS was significantly positively correlated with IEQ (Spearman’s ρ = 0.29, *P* < 0.001; Pearson r = 0.28, *P* = 0.001).

NAIDS showed a significant positive correlation with post-purification IEQ (Spearman’s ρ = 0.29, *P* < 0.001; Pearson r = 0.28, *P* = 0.001; Fig. 1)

**Figure 2.**
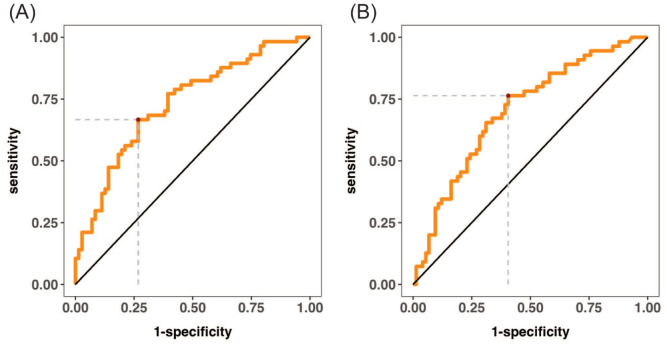
Receiver operating characteristic (ROC) curves for two multivariable logistic regression models predicting successful islet isolation. (a) Model including pre-procurement donor and organ characteristics (BSA, CIT, and pancreas morphology score) yielded an AUC of 0.70 (95% CI: 0.61–0.79, *P* < 0.001). (b) When two intra-procedural variables—digestion temperature (37–38°C) and perfusion temperature (≤14°C)—were added, the model’s AUC improved to 0.788 (95% CI: 0.71–0.87, *P* < 0.001). The diagonal line indicates reference performance (AUC = 0.5).

Multivariate logistic regression identified height >170 cm (odds ratio [OR] = 7.74, 95% CI: 1.42–42.26, *P* = 0.002) and higher BMI (OR = 1.42, 95% CI: 1.03–1.95, *P* = 0.03) as independent predictors of successful islet isolation. Perfusion temperature >14°C was an independent negative predictor (OR = 0.2, 95% CI: 0.05–0.76, *P* = 0.02) (Table 4). A multivariable logistic regression model incorporating BSA, CIT, and morphology score yielded an AUC of 0.70 (95% CI: 0.61–0.79, *P* < 0.001; Fig. 2a). When digestion temperature (37–38°C) and perfusion temperature (≤14°C) were additionally included, the model achieved an AUC of 0.788 (95% CI: 0.71–0.87, *P* < 0.001; Fig. 2b).

**Table 4.** Logistic regression analyses for the prediction of successful islet isolation.

	Univariate		Multivariate	
	OR (95% CI)	*P* value	OR (95% CI)	*P* value
Age (years)	1.00 (0.97–1.03)	0.77		
Weight (kg)	1.02 (1.01–1.04)	0.004	0.86 (0.69–1.07)	0.18
Height	1.04 (1.00–1.08)	0.053		
*<170 cm*	Reference			
*≥170 cm*	3.03 (1.41–6.52)	0.005	7.74 (1.42–42.26)	0.02
BMI	1.07 (1.02–1.13)	0.01	1.42 (1.03–1.95)	0.03
BSA	9.52 (2.24–40.56)	0.002	0.001 (0–223)	0.4
Maximum lipase (U/L)	1.00 (1.00–1.01)	0.12		
Packed tissue volume (ml)	1.06 (1.02–1.10)	0.003	1.03 (0.98–1.08)	0.23
Perfusion temperature				
*4–14°C*	Reference			
*>14°C*	0.21 (0.07–0.59)	0.003	0.20 (0.05–0.76)	0.02
Digestion temp (phase 1)				
*Not strictly control*	Reference			
*>50% digestion time within 37–38°C*	2.75 (1.23–6.16)	0.01	2.30 (0.91–5.82)	0.08
Digestion (phase 1) (min)	0.87 (0.80–0.95)	0.002	0.93 (0.84–1.03)	0.17
NAIDS	1.05 (1.02–1.08)	<0.001	1.03 (0.99–1.07)	0.16

OR, odds ratio; CI, confidence interval; BMI, body mass index; BSA, body surface area; OPT, organ procurement team; BGL, blood glucose level; NAIDS, North American Islet Donor Score; PCV, packed cell volume; temp, temperature. 400K, 400,000; ns, nonsignificant. A value of *P* < 0.05 was considered significant.

### Clinically intended vs research-intended isolations

All 20 clinical-intended isolations met the CITC age criteria (15–65 years). Among the 113 research-intended isolations, nine donors were outside this age range and were excluded, leaving 104 for comparison. The results are presented in Supplementary Table S1. Compared with the research-intended group, the clinically intended group had significantly younger donors (mean 39.7 years vs 47.4 years, *P* = 0.01), higher body weight (mean 104.8 vs 92.1 kg, *P* = 0.03), larger BSA (mean 2.2 vs 2.0 m^2^, *P* = 0.01), and higher NAIDS (median 87 vs 69, *P* = 0.02). The clinical-intended group also had a lower proportion of white donors (64.7% vs 84.9%, *P* < 0.001) and a higher proportion of donors with insulin therapy (76.5% vs 48.1%, *P* = 0.03) and a smoking history (n = 14 (27.8%) vs n = 46 (54.6%), *P* = 0.04).

Procedurally, the clinically intended group had more efficient digestion (86% vs 80%, *P* = 0.002), higher PTV (median 45 mL vs 40 mL, *P* = 0.003), and more consistent digestion temperature control (37–38°C, 78.9% vs 40.7%, *P* = 0.02). No independent predictors emerged from multivariate analysis (Supplementary Table S2).

Of the 20 clinically intended isolations, 16 (80%) proceeded to islet transplantation, as their islet yield exceeded the minimum threshold required for clinical application. Compared to non-transplanted cases, transplanted isolations had significantly greater donor height (median 183 vs 166 cm, *P* = 0.003), larger BSA (median 2.2 vs 2.0 m^2^, *P* = 0.01), higher NAIDS (median 86 vs 63, *P* = 0.02), and longer relative phase 2 digestion time (62.8% vs 48%, *P* = 0.02) (Table 5). Due to the small sample size, logistic regression was not performed on this subgroup.

**Table 5.** Significant differences between transplant and non-transplant groups within clinically intended islet isolations.

	Transplant (n = 16)	Non-transplant (n = 4)	*P*	Effect size
Height (cm)	183 (171–185)	166 (161–169)	0.003	0.6
BSA (m^2^)	2.2 (2.1–2.4)	2.0 (1.8–2.0)	0.01	0.55
NAIDS	86 (70–88)	63 (57–65)	0.02	0.5
Packed tissue volume (ml)	46.5 (45–50)	25 (20.5–37)	0.002	0.65
Digestion time (phase 2, %)	62.8 (59.5–67.7)	48 (45.2–51.3)	0.02	0.66
IEQ ≥400K, n (%)	15 (93.8)	0 (0)	0.004	0.8

Categorical variables were analyzed by Fisher exact test. Continuous variables were analyzed by Mann–Whitney U test. Continuous variables were displayed as median (IQR). IQR, interquartile range; BSA, body surface area; NAIDS, North American Islet Donor Score; IEQ, islet equivalent number. A value of *P* < 0.05 was considered significant.

## Discussion

In this retrospective analysis of 133 human islet isolations, both donor characteristics and procedural parameters were found to influence post-purification islet yield. Elevated BMI was an independent predictor of successful isolation, whereas perfusion temperatures exceeding 14°C were associated with failure. In addition, in exploratory post hoc analysis, donor height ≥170 cm appeared to be associated with a higher likelihood of success; however, this cutoff was not pre-specified and should be interpreted with caution. In addition, we found that tightly regulated digestion temperatures and increased relative duration of phase 2 digestion contributed to higher islet yield. These findings underscore the importance of optimizing both donor selection and procedural consistency to maximize islet recovery to meet the dose requirement for clinical transplantation.

Our data support previous studies showing a positive association between donor anthropometrics and islet yield^5,10,19^. In particular, greater BMI and body weight have been linked to increased pancreatic volume and fat infiltration, which may facilitate enzymatic digestion and islet release^27–30^. Consistent with previous reports, we found that BSA correlated modestly with trimmed pancreas weight (Spearman rho = 0.322, *P* < 0.001)^29^. Interestingly, although body weight, BMI, and BSA were significantly higher in the successful group, trimmed pancreas weight did not differ between groups. This discrepancy may reflect variability introduced during organ procurement and trimming or indicate that pancreatic composition (e.g., fat content, fibrosis) rather than absolute organ weight plays a more important role in determining islet isolation outcomes.

The NAIDS remains a valuable donor-selection tool, as we observed a strong correlation between NAIDS and post-purification yield^5,24^. However, NAIDS was not an independent predictor in our multivariate model, likely due to the lack of inclusion of intraprocedural variables^30^. Similarly, our pancreas morphology scoring system did not show a predictive value, potentially due to inter-observer variability or confounding with other donor characteristics. These findings highlight the limitations of relying solely on donor profiles and underscore the value of incorporating real-time procedural data into yield-prediction models.

Although CIT is widely recognized as a key determinant of islet isolation outcomes^22,31,32^, its lack of predictive value in our study may be explained by our strict adherence to a CIT limit of less than 12 h, consistent with CITC guidelines^8^. By minimizing variability in this parameter, the discriminatory impact of CIT on isolation outcomes was reduced. Prior studies have proposed variable thresholds for CIT-associated risk, ranging from 8 to over 14 h, and have suggested that lukewarm ischemia time^33^—the interval between aortic cross-clamping and pancreatectomy—may be a more sensitive predictor of isolation success^34,35^. Consistent with Nano et al.’s^10^ findings, in our standardized setting, the limited variation in CIT likely reduced its discriminatory value, underscoring the importance of minimizing ischemic exposure as a baseline requirement rather than a predictive variable.

Perfusion temperature emerged as a critical procedural determinant of isolation success. Although the protocol targeted 4–14°C, retrospective review revealed that a subset of cases experienced elevations above 14°C for ≥50% of the perfusion duration due to procedural deviation. Islet isolation is a technically demanding process, and even minor deviations during any step can affect outcomes. Perrier et al.^30^ highlighted this complexity, identifying 230 risks across 10 major workflow steps, with 72% attributed to human error, particularly during enzyme perfusion and digestion phases. Temperature elevations during perfusion likely reflect the technical demands of this stage, where multiple parallel tasks—such as enzyme-recirculation chamber cooling system oversight and continuous monitoring of perfusion flow and pressure—must be coordinated concurrently, which may allow temperature to drift despite initial tight control.

Cases exceeding 14°C for ≥50% of the perfusion duration demonstrated significantly poorer islet yields, and this association remained an independent predictor of isolation failure in multivariate analysis. Although no peer-reviewed data define 14°C as a strict cutoff during the enzyme perfusion stage, our finding that perfusion temperatures >14°C are associated with a higher risk of isolation failure suggests that even modest warming during perfusion may promote pancreatic edema and metabolic stress and thereby compromise islet release and viability, in line with prior work emphasizing strict hypothermic preservation of the pancreas^36^.

These findings underscore that strict thermal regulation is essential not only at perfusion initiation but throughout the entire perfusion period.

Furthermore, our results highlight the importance of tight thermal regulation during phase 1 digestion. Although current guidelines allow a range of 32–38°C, our data show improved outcomes when temperatures were consistently maintained between 37°C and 38°C. This finding reinforces the need for precision in thermal control during enzymatic digestion to minimize islet fragmentation and optimize tissue disaggregation^37,38^.

Our multivariable logistic regression model incorporating BSA, CIT, and pancreas morphology score yielded an AUC of 0.70, indicating moderate discriminative ability for successful isolations based solely on pre-acceptance factors. These three variables provide a simple and practical model compared with composite donor scoring systems such as NAIDS, which incorporate more than 10 donor variables. When two isolation procedural variables—digestion temperature and perfusion temperature—were added, the model’s discriminative ability improved to an AUC of 0.788. Although perfusion and digestion temperatures are not available at the time of organ acceptance, the findings underscore the importance of strict process control and may inform optimization of isolation techniques. Notably, enzyme type and dose were not associated with isolation outcomes in our cohort, suggesting that operator technique and process control may have a greater influence under standardized protocols^19,24,39^.

In the subset of clinically intended isolations, favorable outcomes were associated with higher NAIDS, greater BSA, improved digestion efficiency, and a longer relative duration of phase 2 digestion. There are currently no standardized guidelines regarding the total digestion time or the optimal ratio between phase 1 and phase 2 digestion durations. The transition from phase 1 to phase 2 is typically guided by microscopic assessment criteria, including the presence of more than 45 free islets, a free islet fraction exceeding 50%, and a fragmented islet proportion below 10%. Our finding that a longer relative phase 2 duration was associated with success may have several explanations. One possibility is that more efficient phase 1 digestion, dependent on enzyme potency and tissue quality, liberates islets earlier, allowing a longer phase 2 period under milder enzymatic conditions without causing overdigestion. Alternatively, this association may simply reflect the need to process a larger PTV, which prolongs phase 2 digestion. Further studies are required to disentangle these possibilities and to define evidence-based guidelines for digestion timing.

Limitations of this study include the small number of clinically intended isolations (n = 20), which limits statistical power for multivariate analyses within this subgroup. In addition, this retrospective analysis was limited to isolations performed through 2021, and data from subsequent years were not available for inclusion in the present study. We also did not assess post-transplant graft function or long-term clinical outcomes, which will be addressed in future studies. Nonetheless, the use of a consistent isolation team and protocol over a 15-year span strengthens the internal validity of our findings.

In summary, our study identifies key donor and procedural factors that impact human islet isolation yield. Donor anthropometrics, particularly height and BMI, and procedural variables, including perfusion and digestion temperature, play a critical role in determining isolation success. These insights may help refine selection criteria and protocol parameters to maximize yield and increase the likelihood that an isolation provides sufficient islets to meet clinical dose requirements.

## Conclusion

Donor height and BMI are independent predictors of successful human islet isolation, while elevated perfusion temperature negatively impacts yield. Precise thermal control during phase 1 digestion is essential for efficient early tissue dissociation, allowing an adequately extended phase 2 to maximize islet release and overall yield. Optimizing both donor selection and controlling intra-procedural variables can improve islet yield and maximize the likelihood of meeting the clinical transplantation dose requirement.

## Supplemental Material

sj-docx-1-cll-10.1177_09636897261433325 – Supplemental material for Human islet isolation optimization: Insights from donor and isolation procedural factors Supplemental material, sj-docx-1-cll-10.1177_09636897261433325 for Human islet isolation optimization: Insights from donor and isolation procedural factors by Qin Yang, Yinsheng Xi, Zhihong Yang, Hongping Deng, Zhenjuan Wang, Guoping Li, Kerry Augusta, Shimul Shah, James F. Markmann and Ji Lei in Cell Transplantation
