# Supplementary material for: Human islet isolation optimization: Insights from donor and isolation procedural factors
Source: Cell Transplant. 2026 Mar 24;35:09636897261433325. doi: 10.1177/09636897261433325 (PMC13018692; doi:10.1177/09636897261433325)
Supplement: sj-docx-1-cll-10.1177_09636897261433325 – Supplemental material for Human islet isolation optimization: Insights from donor and isolation procedural factors [file sj-docx-1-cll-10.1177_09636897261433325.docx]

**Supplementary Information**

**Human Islet Isolation Optimization: Insights from Donor and Isolation Procedural Factors** Qin Yang^1^, Yinsheng Xi^2^, Hongping Deng^2^, Zhihong Yang^2^, , Zhenjuan Wang^2^, Guoping Li^2^, James F. Markmann^1^, Ji Lei^1.2^

Table S1. Difference in donor characteristics and islet product outcome parameters between clinical and research intend isolation

|  | Clinically intended  (n=20) | Research-intended  (n=104) | *p* |  |
| --- | --- | --- | --- | --- |
| Age, years | 39.7$\pm$12.4 | 47.4$\pm$12.2 | 0.01 |  |
| Weight (kg) | 104.8$\pm$18.5 | 92.1$\pm$24.4 | 0.03 |  |
| Height (cm) | 178.3$\pm$9.8 | 172.6$\pm$9.3 | 0.02 |  |
| BSA(m^2^) | 2.2$\pm$0.2 | 2.0$\pm$0.3 | 0.01 |  |
| BMI | 32.9$\pm$4.0 | 31.1$\pm$7.5 | 0.31 |  |
| OPT (distant), n (%) | 17 (89.5) | 106 (93) | 0.59 |  |
| Race (white/Caucasian), n (%) | 11 (64.7) | 90 (84.9) | <0.001 |  |
| Sex (male) | 11 (57.9) | 68 (61.3) | 0.78 |  |
| Death cause (cerebrovascular), n (%) | 10 (52.6) | 54 (47.8) | 0.92 |  |
| Type of vasopressor (none), n (%) | 1 (7.1) | 28 (30.1) | 0.11 |  |
| Insulin therapy, n (%) | 13 (76.5) | 50 (48.1) | 0.03 |  |
| Smoking, n (%) | 5 (27.8) | 59 (54.6) | 0.04 |  |
| Alcohol, n (%) | 13 (72.2) | 76 (70.4) | 0.87 |  |
| Hypertension, n (%) | 7 (38.9) | 37 (34.3) | 0.70 |  |
| NAIDS | 87 (77-88) | 69 (64-79) | 0.02 |  |
| Morphology score | 9 (7-11) | 8 (7-10) | 0.28 |  |
| Minimum BGL (mg/dL) | 122 (101-129) | 106 (90-121) | 0.72 |  |
| Maximum BGL (mg/dL) | 215 (187-264) | 225 (185-277) | 0.79 |  |
| Maximum lipase (U/L) | 45 (18-200) | 28 (19-70) | 0.45 |  |
| Maximum Amylase (U/L) | 184 (50-309) | 86 (55-187) | 0.27 |  |
| Acute illness time (day) | 1.0 (1.0-1.0) | 1.0 (1.0-3.0) | 0.01 |  |
| Brain death time (hour) | 16 (13.3-17.8) | 19.3 (13.0-29.2) | 0.07 |  |
| Cold ischemia time (hour) | 8.2 (7.6-11.3) | 8.6 (6.8-9.8) | 0.80 |  |
| Trimmed pancreas (g) | 97 (83-100) | 96 (82-102) | 0.38 |  |
| Undigested Tissue (g) | 12 (10-18) | 19 （13-29.5） | 0.30 |  |
| Digested tissue percentage (%) | 86 （81-89） | 80 （73-86） | 0.002 |  |
| Enzyme type (Serva), n (%) | 15 (88.2) | 52 (46.8) | 0.001 |  |
| Preservation (UW), n (%) | 18 (100) | 79 (84.9) | 0.12 |  |
| Packed tissue volume (ml) | 45 (40-50) | 40 (30-48) | 0.003 |  |
| Digestion time (phase 1, min) | 15 (11-15) | 17 (13-19) | 0.12 |  |
| Digestion time (phase 2, %) | 60 (50.2-64.6) | 50.6 (46.9-60.5) | 0.01 |  |
| Perfusion temperature (≥14℃), n (%) | 1 (5.3) | 27 (24.8) | 0.07 |  |
| Digestion temp (37-38℃), n (%) | 15 (78.9) | 46 (40.7) | 0.02 |  |
| Purified IEQ (≥400,000), n (%) | 15 (78.9) | 41(39.4) | <0.001 |  |

Categorical variables were analyzed by Fisher exact test; continuous variables were analyzed by Mann–Whitney U-test. Continuous variables were displayed as median (IQR). IQR, interquartile range; AA, Africa-American; POC, people of color; BSA, body surface area; NAIDS, North American Islet Donor Score; IEQ, islet equivalent number; NP, Neutral Protease; COL, collagenase; IPN, islet particle number; IEQ, islet equivalent number; PCV, packed cell volume; 400K, 400,000; temp, temperature; ns, nonsignificant. A value of p<0.05 was considered significant.

Table S2. Logistic regression analyses predicting clinical transplant outcomes in clinically intended islet isolation.

|  | *Univariate* | |
| --- | --- | --- |
|  | OR (95%CI) | *P* Value |
| Age (years) | 1.05 (1.01-1.10) | 0.02 |
| Weight (kg) | 0.98 (0.96-1.00) | 0.04 |
| Height | 0.94 (0.88-0.99) | 0.02 |
| BSA | 0.08 (0.01-0.53) | 0.008 |
| BMI | 0.97 (0.91-1.03) | 0.31 |
| NAIDS | 0.96 (0.93-1.00) | 0.03 |
| Acute illness duration (day) | 1.7 (0.96-2.95) | 0.07 |
| History of insulin therapy | 0.29 (0.09-0.93) | 0.04 |
| History of smoking | 3.13 (1.04-9.40) | 0.04 |
| Digested tissue (%) | 0.97 (0.94-1.0) | ns. |
| Packed tissue volume (ml) | 0.93（0.89-0.99） | 0.02 |
| Perfusion temp (≥14℃) | 0.17 (0.02-1.32) | 0.09 |
| Digestion temp (phase1, 37-38℃) | 5.46 (1.70-17.51) | 0.004 |
| Digestion time (phase 2, %) | 0.94 (0.89-0.99) | 0.01 |
| Enzyme (Serva) | 8.5 (1.86-38.98) | 0.006 |

OR, odds ratio; CI, confidence interval; BMI, body mass index; BSA, body surface area; temp, temperature; OPT, organ procurement team; BGL, blood glucose level; NAIDS, North American Islet Donor Score; PCV, packed cell volume; temp, temperature. 400K, 400,000; ns, nonsignificant. A value of p<0.05 was considered significant.
